# The 3D Printing of Novel Honeycomb–Hollow Pyramid Sandwich Structures for Microwave and Mechanical Energy Absorption

**DOI:** 10.3390/polym15244719

**Published:** 2023-12-15

**Authors:** Quan Li, Zhicheng Wang, Xueyang Wang, Yang Wang, Jian Yang

**Affiliations:** 1 Jiangsu Collaborative Innovation Center for Advanced Inorganic Function Composites, Nanjing Tech University, Nanjing 211816, China; wangyang_89@163.com (Y.W.); yangjian1976@163.com (J.Y.); 2 College of Materials Science and Engineering, Nanjing Tech University, Nanjing 211816, China; wzc99994@163.com (Z.W.); 1275956819nz@gmail.com (X.W.)

**Keywords:** honeycomb–hollow pyramid metastructure, microwave absorption, mechanical energy absorption, PLA/CNT

## Abstract

Honeycomb sandwich (HS) structures are important lightweight and load-bearing materials used in the aerospace industry. In this study, novel honeycomb–hollow pyramid sandwich (HPS) structures were manufactured with the help of fused deposition modeling techniques using PLA and PLA/CNT filaments. The microwave and mechanical energy absorption properties of the HPS structures with different geometry parameters were studied. Compared with the HS structure, the HPS structure enhanced both microwave absorption and mechanical properties. The HPS structures possessed both broadband and wide-angle microwave absorption characteristics. Their reflection loss at 8–18 GHz for incident angles of up to 45° was less than −10 dB. As the thickness of the hollow pyramid increased from 1.00 mm to 5.00 mm, the compressive strength of the HPS structure increased from 4.8 MPa to 12.5 MPa, while mechanical energy absorption per volume increased from 2639 KJ/m^3^ to 5598 KJ/m^3^. The microwave absorption and compressive behaviors of the HPS structures were studied.

## 1. Introduction

Honeycomb sandwich (HS) structures are essential as load-bearing structures in aircraft due to their light weight, high specific strength, high stiffness, and high specific impact energy absorption properties [1,2,3]. Over the past decade, the need to protect aircraft from radar detection has promoted the development of microwave-absorbing honeycomb sandwich structures (MAHS) [4,5,6,7]. Filling the honeycomb cavities with microwave-absorbing foams or coating the honeycomb side walls with microwave-absorbing agents are conventional methods for producing MAHS [8,9,10]. However, the MAHS are still plagued by their narrow effective absorption bandwidth, weight increment, or complicated processing procedure [11].

Meta-material absorbers (MMA) have artificial periodic structures that allow for a high degree of control over the microwave absorption properties through geometry design [12,13,14,15]. Various three-dimensional meta-structures have been successfully constructed to improve microwave absorption properties [15,16,17]. Typical configurations are gradient structures, such as multi-step or pyramidal structures [15,18]. Due to the impedance matching condition and multiple microwave attenuation mechanisms, these gradient meta-structures exhibit strong and broadband microwave absorption properties. However, because of their unusual shapes, they are susceptible to damage at the apex area of the gradient structures. Therefore, these meta-structures are difficult to use directly as load-bearing structures.

Combining an HS structure with MMA (HS-MMA) could be an effective method for fabricating structures with both excellent load-bearing and microwave-absorbing properties. In recent years, the modification of honeycomb structures with MMA has emerged as a method for improving microwave absorption properties. Huang et al. inserted an H-shape meta-material unit into the side walls of a honeycomb, which expanded the effective absorption bandwidth from 8.3 GHz to 15.7 GHz [19]. Ghosh et al. painted square-loop resistive meta-structures on the front surface of PLA honeycomb [20]. The structure had a broadband absorption over the frequency range from 5.5 GHz to 16.9 GHz. He et al. incorporated a frequency-selective surface (FSS) into a patterned honeycomb absorber [21]. The resulting structure had an effective absorption bandwidth of 2.89–18 GHz. According to the aforementioned reports, the HS-MMA structures are composed of complex-shaped microwave-absorbing and dielectric materials. The assembly of these materials into intricate HC-MMA structures is hard and time-consuming.

The technology of 3D printing is the state of the art for fabricating complex structures. Therefore, 3D printing has extraordinary advantages for making HS-MMA structures [22,23]. In this paper, a novel microwave-absorbing sandwich structure was designed by incorporating hollow pyramid meta-structures into the honeycomb cavities of an HS structure. Due to its affordability and user-friendly nature, the fusing deposition modeling (FDM) apparatus was chosen to construct the HS-MMA structures. This technique offers the advantages of low warpage, superior mechanical properties, and unique functional properties in the fabricated samples. The polylactic acid/carbon nanotube (PLA/CNT) filaments were used to integrally fabricate a honeycomb–hollow pyramid core with a base panel in one step. This structure was then easily attached to the PLA front panel. The results indicate that incorporating pyramid meta-structures into the HS structure not only improved the microwave absorption properties but also the compressive energy absorption properties. This study provides a novel and straightforward method for fabricating lightweight structures with both good microwave and mechanical energy absorption properties.

## 2. Materials and Methods

### 2.1. Materials Preparation

**Figure 1:**
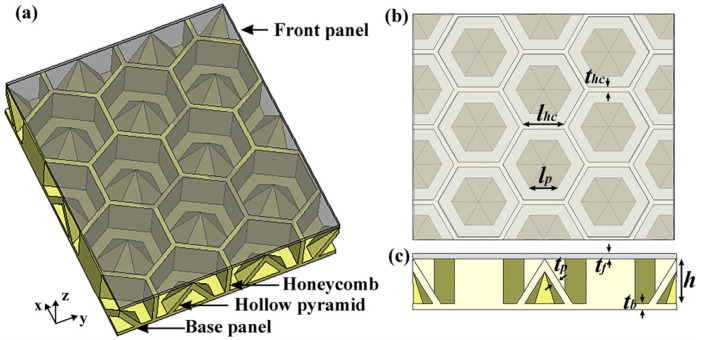
Schematic diagram of (**a**) the HPS structure, (**b**) view in the x-y plane, and (**c**) view in the x-z plane.

PLA and PLA/CNT filaments (Hangzhou Linan Beisen E-commerce Co., Ltd., Hangzhou, China) were used as raw materials. FDM was used to fabricate the structure using a 3D printer (Ender-3, Creality, Shenzhen, China) with a nozzle diameter of 0.4 mm, an infill density of 100%, a printing temperature of 215 °C, and a printing speed of 60 mm/s. Figure 1a displays the schematic diagram of the HPS structure, which is composed of a front panel, a honeycomb–hollow hexagonal pyramid structure core, and a base panel. The front panel is a PLA flat plate, which serves as an impedance matching layer. The honeycomb–hollow pyramid core and base panel are made of PLA/CNT composites, which serve as microwave attenuation structures. As shown in Figure 1b, the geometry parameters of the HPS structure are defined as follows: *l_hc_* and *t_hc_* are the length and thickness of the honeycomb side wall; *l_p_* and *t_p_* are the base side length and thickness of the hollow pyramid; *h* is the height of the honeycomb and hollow pyramid; *t_f_* and *t_b_* are the thicknesses of the front and base panels, respectively.

In order to obtain the relative complex permittivities of PLA and PLA/CNT composites, samples with dimensions of 22.86 × 10.16 × 2.00 mm^3^ and 15.80 × 7.90 × 2.00 mm^3^ were printed for the X and Ku band tests, respectively. The data were imported into CST Microwave Studio (2020), which was used to simulate and optimize the microwave absorption properties of HPS structures. According to optimized geometry parameters, the HPS structures were fabricated with FDM. Samples with 2 × 3 units were printed for the compressive strength test. HPS structures with dimensions of 180.00 mm × 180.00 mm (in length and width) were printed for the reflection loss test. To prepare the HPS structure, the honeycomb–hollow pyramid core and base panel were first printed using PLA/CNT filaments in one step, while the front panel was printed using PLA filament. Then, the front panel was assembled on the honeycomb–hollow pyramid structure using epoxy resin. By comparison, the microwave absorption and mechanical properties of HP structures composed of a PLA front panel, a PLA/CNT honeycomb core, and a PLA/CNT base panel were also studied.

### 2.2. Characterization

**Figure 2:**
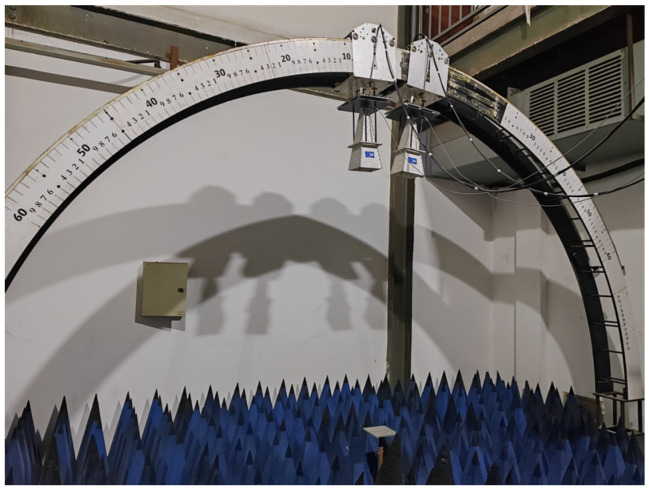
The arch-based reflection loss test system.

The microstructures of PLA/CNT filament and printed samples were examined using a scanning electron microscope (SEM, SU8010, Hitachi, Tokyo, Japan). The compositions of printed samples were analyzed with Raman spectra, which were obtained with a microscope (Horiba Jobin Yvon, Bensheim, Germany) using a laser wavelength of 514 nm. A four-probe apparatus (RTS-9, 4Probes Tech Ltd., Guangzhou, China) was used to measure the electrical conductivity of the printed samples. A vector network analyzer (VNA, MS4644A, Atsugi, Japan) equipped with waveguides (X and Ku bands) was used to obtain the complex permittivity of printed samples. The microwave absorption property of PLA/CNT flat plates was predicted by *RL*, which was obtained according to Equations (1) and (2) based on the metal back-panel model [24].

(1) $$RL=20{log}_{10}(\left|Z_{in}-1\right|/\left|Z_{in}+1\right|)$$

(2) $$Z_{in}=\sqrt{\mu_{r}/\varepsilon_{r}}tanh\left[j2\pi \sqrt{\mu_{r}\varepsilon_{r}}fd/c\right]$$

where *Z_in_* was the normalized input impedance, *c* was the light velocity in vacuum, *f* was the electromagnetic wave frequency, *d* was the sample thickness, *ε_r_* was the relative complex permittivity, and *μ_r_* was the relative complex permeability, respectively. The reflection loss (*RL*) of the HPS structure was measured by an arch-based reflection loss test system (shown in Figure 2) at a frequency range of 8–18 GHz in an anechoic chamber. The compressive strength of HPS structures was measured on a universal test machine (CMT5245, NSS, Shenzhen, China) with a crosshead speed of 2.00 mm/min. The load was applied along the height direction of the structure. The nominal compressive strength is defined as the load divided by the whole cross-section area of the structure.

## 3. Results and Discussion

### 3.1. Microstructure and Electromagnetic Characterization of PLA/CNT Composites

**Figure 3:**
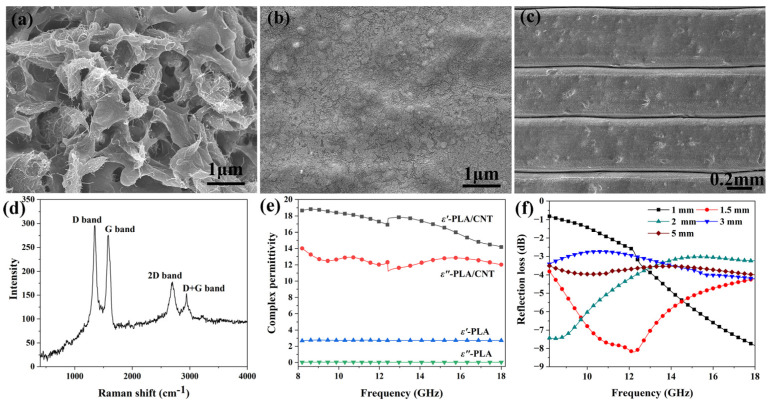
SEM images of (**a**) PLA/CNT filament, (**b**,**c**) printed PLA/CNT composites at high and low magnifications, (**d**) Raman spectra of PLA/CNT composite, (**e**) relative complex permittivities of PLA/CNT and PLA composites, and (**f**) reflection loss of PLA/CNT composite.

Both the material’s intrinsic properties and the structural design determine the microwave absorption and mechanical properties of HPS structures. Therefore, the microstructures and dielectric properties of PLA/CNT filaments and printed flat samples were investigated first. Figure 3a–c shows the microstructures of the as-received PLA/CNT filament and printed samples, respectively. Clearly, the raw PLA/CNT filament has a porous structure. During printing, the PLA/CNT filament completely re-melted, resulting in a dense matrix of the printed sample, as shown in Figure 3b. As indicated in Figure 3c, the printed PLA/CNT composite has a layered structure, which is a typical microstructure for the material fabricated by the 3D printing method. Each layer has an average thickness of around 450 μm. In addition, Figure 3a illustrates the presence of CNT aggregation in the PLA matrix of filaments. Nevertheless, the image reveals that these CNTs are intertwining or linking to form a CNT network within the PLA matrix. As shown in Figure 3d, the sharp D band and G band on the Raman spectrum of the composite indicate well-crystallized CNT in the PLA matrix. As a result, the PLA/CNT composite, whose value reaches 2.5 S/m, has good electrical conductivity.

The dielectric properties of printed PLA and PLA/CNT plates were measured, and the results are shown in Figure 3e. The PLA plate has low real and imaginary permittivity, which are only 2.7 and 0.03, respectively. As a result, the PLA plate is a good impedance-matching material. As shown in Figure 3a, the PLA/CNT composite has rich heterointerfaces between the CNT and PLA matrix, which cause high polarization loss under microwave radiation [25]. In addition, the high electrical conductivity of the PLA/CNT composite indicates that the composite also has a high conduction loss under microwave radiation. Consequently, the PLA/CNT composite has a high intrinsic attenuation capacity for microwaves introduced into the samples, as indicated by its high loss tangent (0.7 at 13 GHz). However, its high permittivities (*ε*′ = 18 and *ε*″ *=* 12.5 at 13 GHz) result in poor impedance matching conditions with free space. As a result, as shown in Figure 3f, the PLA/CNT flat plate has weak microwave absorption properties. The reflection loss at 1.50 mm is only smaller than −4 dB from 8 GHz to 18 GHz, and the minimum value is only −8.3 dB. As the sample thickness is greater than 3.00 mm, the reflection loss is greater than −4 dB from 8 GHz to18 GHz, which indicates more than 40% of microwave energy is reflected by the PLA/CNT composite.

### 3.2. Microwave Absorption Performance of the HPS Structure

**Figure 4:**
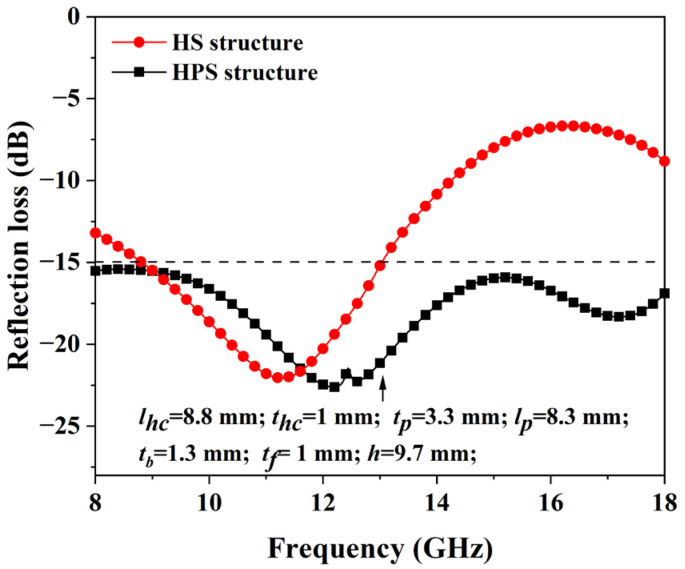
Simulated reflection loss for the HPS and HS structures.

The microwave absorption performance of the HPS structures was optimized by employing the genetic algorithm in CST software (2020). Figure 4 shows the HPS structure’s optimized reflection loss and the corresponding geometry parameters. For comparison, the microwave absorption performance of the HP structure was also simulated. It can be seen from Figure 4 that the reflection loss of the HS structure is less than −10 dB from 8 GHz to 14.2 GHz. Nevertheless, from 14.2 GHz to 18 GHz, the HS structure has poor microwave absorption properties. After incorporating the hollow pyramid into the honeycomb core, the simulated reflection loss of the HPS structure is less than −15 dB from 8 GHz to 18 GHz, indicating more than 96% microwaves could be absorbed by the HPS structure. The results suggest that the incorporated hollow pyramids could effectively enhance the microwave absorption properties of the structure, especially at high frequencies. When compared to other honeycomb–MMA structures [19,20,21], the HPS structures exhibit superior microwave absorption properties in the frequency range of 8–18 GHz.

**Figure 5:**
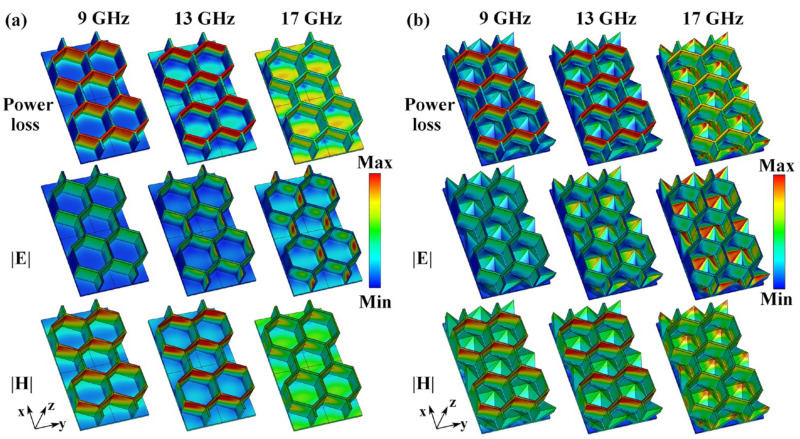
Power loss density, electric field, and magnetic field of (**a**) HS structure and (**b**) HPS structure at 9 GHz, 13 GHz, and 17 GHz.

To determine the microwave absorption mechanisms of HS and HPS structures, the distributions of power loss density, electric field intensity, and magnetic field intensity at 9 GHz, 13 GHz, and 17 GHz were simulated. The results are shown in Figure 5. Since the PLA front panel is an impedance matching layer, its power loss can be ignored. Therefore, the power loss on the PLA front panel is not shown in Figure 5. As shown in Figure 5a, the power loss at low frequencies (9 GHz and 13 GHz) for the HS structure is primarily concentrated on the honeycomb side wall. As the frequency increases to 17 GHz, the power loss density on the side wall decreases, while it increases on the base panel. As a result, microwave absorption at low frequencies occurs primarily in the honeycomb of the HS structure, while absorption at high frequencies occurs primarily on the base panel. However, the power loss density on the base panel is low, resulting in a low microwave absorption property of the HS structure at high frequencies, as shown in Figure 4. At 9 GHz, the electric field of the HS structure is nearly uniformly distributed across the entire honeycomb side walls. As the frequency increases to 17 GHz, the electric field on the side walls and base panel becomes stronger. By comparison, the magnetic fields at 9 GHz and 13 GHz are primarily distributed on the side walls along the y-axis direction. At 17 GHz, the intensity of the magnetic field decreases and concentrates at the corners of the honeycomb side walls. On the contrary, the magnetic field intensity at the base panel is enhanced as the frequency increases from 9 GHz to 17 GHz. It can be seen in Figure 5a that the distribution area of power loss density on the honeycomb wall overlaps with that of the magnetic field. This suggests that the magnetic field plays a dominant role in microwave absorption on the honeycomb side wall [26,27]. This is because, under the oscillating magnetic field, the induced currents build up in the honeycomb side wall. For the base panel, the distribution area of power loss overlaps with both the magnetic field and the electric field, suggesting that both fields contribute to the microwave absorption on the base panels.

**Figure 6:**
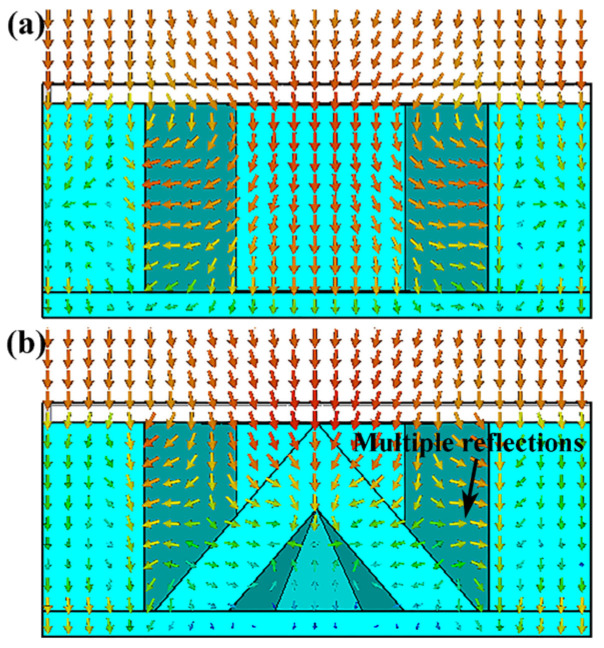
Power flow of (**a**) HP structure and (**b**) HPS structure at 17 GHz. The arrows in the figures are Poynting’s vectors.

Figure 5b shows that, at low frequencies (9 GHz and 13 GHz), the distributions of power loss density, electric field, and magnetic field on the honeycomb side walls of the HPS structure are similar to those of the HS structure. This indicates that the introduction of hollow pyramids does not significantly change the field distribution on the honeycomb side wall. Therefore, in the HPS structure, honeycomb side walls are also the primary structures for low-frequency microwave absorption. As the frequency increases from 9 GHz to 17 GHz, the power loss on the pyramid increases. At 17 GHz, the apex area of the hollow pyramid has a high power loss density. Its distribution area overlaps with both the electric and magnetic fields, indicating that both fields contribute to the microwave absorption of the hollow pyramid. On the other hand, strong absorption also occurs at the corner between the honeycomb side wall and hollow pyramid at 17 GHz. To explain this result, the power flow at 17 GHz in both HS and HPS structures was obtained using CST software (2020). As shown in Figure 6, it is clearly noted that the microwaves are reflected between the hollow pyramid and honeycomb wall and finally propagate towards the corner where they are absorbed. Due to the strong absorption of hollow pyramids and multiple reflections, the HPS structure has better microwave absorption properties at high frequencies than the HS structure.

**Figure 7:**
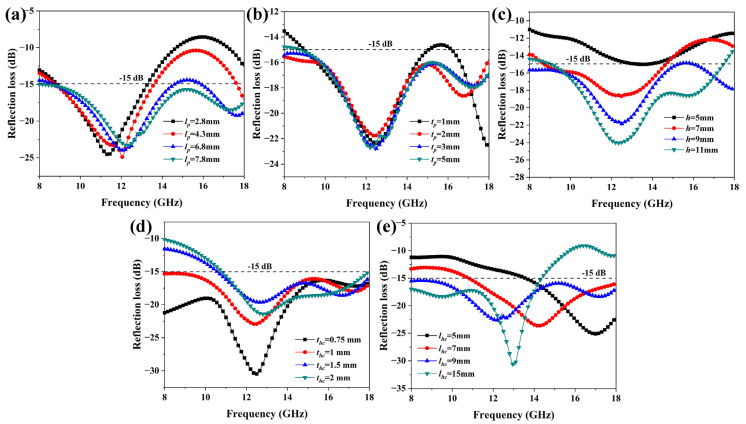
Geometry effects on the microwave absorption of HPS structures: (**a**) *l_p_*, (**b**) *t_p_*, (**c**) *h*, (**d**) *t_hc_*, and (**e**) *l_hc_*.

Both honeycomb and hollow pyramids play a critical role in the HPS structure’s microwave absorption, as demonstrated by the previous results. Consequently, it is necessary to elucidate the effects of geometry parameters (*l_p_* and *t_p_* of the pyramid; *h*, *t_h_*, and *l_h_* of the honeycomb) on the microwave absorption properties of the HPS structure. As shown in Figure 7a, the *l_p_* has a remarkable effect on the microwave absorption of the HPS structure at high frequencies. The increase in *RL* of the HPS as *l_p_* decreases indicates deteriorating microwave absorption properties. This is primarily due to the fact that decreasing *l_p_* reduces multiple reflections between the honeycomb side walls and hollow pyramids. It is interesting to note that the *t_p_* has a very small effect on the microwave absorption properties of the HPS structure when it is in the range of 2.00–5.00 mm. As a result, it is possible to decrease the weight of the HPS structure without sacrificing its microwave absorption performance by reducing *t_p_*. As *h* increases, the propagation pathway of microwaves in the honeycomb increases. On the other hand, the multi-reflection of microwaves between the honeycomb wall and pyramid is also enhanced as *h* increases. As a result, as shown in Figure 7c, the microwave absorption properties of the HPS structure increase as *h* increases. As stated in the aforementioned section, the low-frequency microwaves are primarily absorbed by the honeycomb side walls. Therefore, variations in the *t_h_* mainly affect the microwave absorption properties at low frequencies. As shown in Figure 7d, the microwave absorption properties of the SHP structure at low frequencies increase as *t_hc_* increases. As shown in Figure 7e, the absorption peaks have a red shift as *l_hc_* increases, which is consistent with the results reported in the literature [23]. It can be seen from Figure 7 that the HPS structure has broadband microwave absorption properties even when the geometry parameters vary over significant ranges. This brings convenience and flexibility to implementing the HPS structure on an aircraft. For example, the weight of aircraft can be reduced by changing the height, thickness, or length of hollow pyramids and honeycomb side walls in the HPS structure, which still has broadband microwave absorption properties. In addition, it is well known that the mechanical properties of the honeycomb structure have a positive relationship with *t_p_* and *t_hc_*, while they have a negative relationship with *l_p_* and *l_hc_* [28]. Therefore, by tailoring their geometry parameters, the HPS structures could be given both broadband absorption and improved mechanical properties.

**Figure 8:**
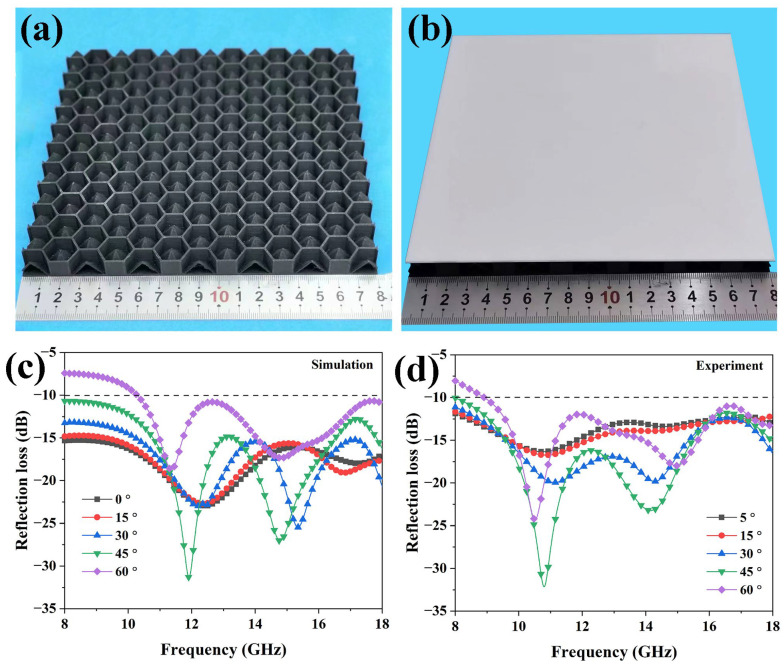
(**a**) The 3D printed honeycomb–hollow pyramid core with a base panel, (**b**) the HPS structures, (**c**) simulated reflection loss at different incident angles, and (**d**) measured reflection loss at different angles.

In order to verify the microwave absorption property of the HPS structure, samples with optimized geometry parameters (displayed in Figure 4) were printed and assembled into the sandwich structure shown in Figure 8b. The microwave absorption properties of the optimized HPS structure were simulated and measured at various incident microwave angles. Figure 8c,d reveal that the overall trend of the simulated results corresponds well with the measured results. The slight red shifts in absorption peaks between simulated and measured results are likely due to geometry errors in the printed samples [29]. As shown in Figure 8c,d, the two absorption peaks have red shifts as the incident angle increases. When the incident angle is less than 45°, the absorption intensity increases as the incident angle increases, and the *RL* is less than −10 dB over the entire 8–18 GHz range. As the incident microwave angle further increases to 60°, the *RL* is smaller than −10 dB from 10.2 GHz to 18 GHz. The above results indicate that the HPS structure has broadband microwave absorption properties over a wide range of incident angles.

### 3.3. Mechanical Property of Honeycomb-Pyramid Structure

**Figure 9:**
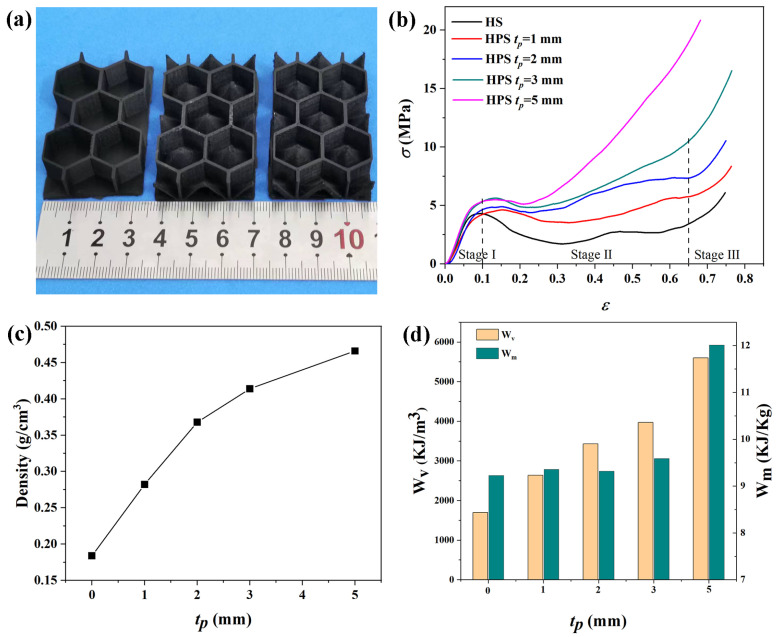
(**a**) Samples for the compressive strength test; (**b**) stress–strain curves, (**c**) sample densities, and (**d**) the mechanical energy absorption of the structures at different *t_p_*.

To study the effects of *t_p_* on the mechanical properties, honeycomb–hollow pyramid structures with varying *t_p_* were fabricated, and some samples are shown in Figure 9a. For comparison, the HP samples were also printed. The out-of-plane compressive strengths of the HPS and HP structures were measured. Figure 9b displays the strain (*ε*)–stress (*σ*) curves of the structures. The compression behavior of the structures has three stages. When the strain is less than 0.1 (stage I), all the samples exhibit similar compression behavior. This is because the honeycomb side wall is the primary load-bearing structure at this stage. As the strain increases from 0.1 to 0.65 (stage II), the stress of the HP structure rapidly decreases and reaches a plateau stage, which is caused by the buckling of the honeycomb side walls. For HPS structures, the plateau stage is shortened, and stress rises quickly. Figure 9b reveals that the nominal compressive strength of the HPS structure increases as *t_p_* increases. At a strain of 0.5, the nominal compressive strength of the HPS structure with *t_p_* = 3.00 mm is 7.5 MPa, which is nearly three times larger than that of the HS structure. This is because the pyramid is the main load-loading structure in HPS at this stage. Stage III (*ε* > 0.65) is the densification stage, in which the buckling structures come into contact with one another, leading to a rapid increase in compression strength. Therefore, the incorporation of hollow pyramids into honeycomb cavities could effectively enhance the compression strength of the sandwich structure.

Mechanical energy absorption is another important parameter for assessing the mechanical properties of load-bearing structures. The mechanical energy absorption per unit volume (*W_v_*) and per unit mass (*W_m_*) can be obtained by the following equations [30,31,32]:

(3) $$Wv=\int_{0}^{\varepsilon_{D}}\sigma d\varepsilon$$

(4) $$W_{m}=W_{v}/\rho$$

where *ρ* is the bulk density of the structure and *ε_D_* is the densification strain [31]. According to Figure 9b, *ε_D_* is 0.65 for HS and HPS structures in this study. Figure 9c shows the bulk density, which was calculated by dividing the total mass of the structure by its whole volume. The HS structure has a low bulk density of 0.18 g/cm^3^. The bulk density of the HPS structure increases from 0.28 to 0.46 g/cm^3^ as *t_p_* increases from 1.00 mm to 5.00 mm. This result suggests that the hollow structure effectively decreases the bulk density of the structure. Figure 9d displays the *W_v_* and *W_m_* for the HS and HPS structures. The *W_v_* and *W_m_* for the HS structure are 1698 KJ/m^3^ and 9.2 KJ/Kg, respectively. By comparison, the HPS structure has a higher *W_v_*, which increases from 2639 KJ/m^3^ to 5598 KJ/m^3^ as *t_p_* increases from 1.00 mm to 5.00 mm. As *t_p_* increases from 1.00 mm to 3.00 mm, the *W_m_* of the HPS structure only increases from 9.3 to 9.6 KJ/Kg. However, as *t_p_* increases to 5.00 mm, *W_m_* increases to 12.1 KJ/Kg. The above results indicate that the HPS structure not only has good microwave absorption properties but also has good mechanical energy absorption properties. Therefore, the HPS structures can be used as lightweight, load-bearing, and microwave absorbing components in stealth aircraft. The novel structures designed in this study can also be fabricated with stronger polymer composites, such as ABS/CNT, to improve their mechanical properties.

## 4. Conclusions

Novel sandwich structures composed of a PLA front panel, a PLA/CNT honeycomb-pyramid core structure, and a PLA/CNT base panel were designed and fabricated with the aid of the FDM technique. The microwave and mechanical energy absorption properties of a structure with variations in geometry parameters were explored. The sandwich structure with optimized geometry parameters has both broadband and wide-angle microwave absorption properties. The reflection loss is less than −10 dB from 8 to 18 GHz at an incident angle up to 45°. In the frequency range of 8–18 GHz, the honeycomb side walls of the designed structure mainly absorb microwaves in the low-frequency band, while the hollow pyramids mainly absorb microwave in the high-frequency band. The sandwich structure has three stages of compression behavior. The honeycomb is the main loading structure in the first stage, while the hollow pyramid is the main loading structure in the second stage. As the thickness of the hollow pyramid increases, the compression strength of the structure increases, resulting in an increase in mechanical energy absorption. The designed novel structures can be utilized as lightweight, load-bearing, and microwave-absorbing structure components in aircraft.

## Data Availability

The data presented in this paper are available on request from the corresponding author.
